# Relation between initial hypothermia, course of the hypothermia and mortality in patients with septic shock: a post-hoc analysis of the SEPSISPAM randomized trial

**DOI:** 10.1016/j.aicoj.2026.100051

**Published:** 2026-03-18

**Authors:** Louis Bordeau, Valérie Seegers, Julien Demiselle, Frédérique Schortgen, Fabien Grelon, Bruno Mégarbane, Nadia Anguel, Jean-Paul Mira, Pierre-François Dequin, Soizic Gergaud, Nicolas Weiss, François Legay, Yves Le Tulzo, Marie Conrad, Remi Coudroy, Frédéric Gonzalez, Christophe Guitton, Fabienne Tamion, Jean-Marie Tonnelier, Jean Pierre Bedos, Thierry Van Der Linden, Antoine Vieillard-Baron, Eric Mariotte, Gaël Pradel, Olivier Lesieur, Jean-Damien Ricard, Fabien Hervé, Damien du Cheyron, Claude Guerin, Alain Mercat, Jean-Louis Teboul, Peter Radermacher, Pierre Asfar, Nicolas Fage

**Affiliations:** a Department of Medical Intensive Care, University Hospital of Angers, Angers, France; b Biometry Department Biométrie, Western Cancer Institute, Paul Papin Center, Angers, France; c Department of Intensive Care (Service de Médecine Intensive – Réanimation), Hôpital de Hautepierre, University Hospital of Strasbourg, Strasbourg, France; d INSERM (French National Institute of Health and Medical Research), UMR 1260, Regenerative Nanomedicine (RNM), FMTS (Fédération de Médecine Translationnelle de Strasbourg), University of Strasbourg, Strasbourg, France; e Intercommunal Hospital Center, Department of Adult Intensive Care, Créteil, France; f Medical and Surgical Intensive Care Unit, Le Mans Hospital, Le Mans, France; g Department of Medical and Toxicological Critical Care, Lariboisière Hospital, Paris University, INSERM UMRS-1144, Paris, France; h Department of Medical Intensive Care, Bicêtre University Hospital, AP-HP, Paris-Saclay University, Le Kremlin Bicêtre, France; i Department of Medical Intensive Care, Cochin Hospital, Assistance Publique - Hôpitaux de Paris, Paris, France – University of Paris Cité, Paris, Franc - Institut Cochin, INSERM U1016, CNRS UMR 8104, University of Paris Cité, France; j Department of Medical Intensive Care, Tours University Hospital, Tours, France; k Department of Surgical Intensive Care, University Hospital of Angers, Angers, France; l Department of Medical Intensive Care, Georges Pompidou European Hospital, Assistance Publique – Hôpitaux de Paris, University of Paris, Paris, France; m Medical and Surgical Intensive Care Unit, Saint Brieuc Hospital, Saint Brieuc, France; n Department of Infectious Diseases and Medical Intensive Care, Rennes University Hospital, Rennes, France; o Department of Medical Intensive Care, Nancy University Hospital, Nancy, France; p Department of Medical Intensive Care, INSERM CIC-1402, IS-ALIVE Research Group, University of Poitiers, CHU Poitiers, Poitiers, France; q Department of Medical and Surgical Intensive Care, Avicenne Teaching Hospital, Bobigny, France; r Department of Medical Intensive Care, Nantes University Hospital, Nantes, France; s Univ Rouen Normandie, Inserm, ENVI UMR 1096, Department of Medical Intensive Care, F-76000 Rouen, France; t Department of Medical Intensive Care, Brest University Hospital, Brest, France; u Intensive Care Unit, Versailles Hospital, Le Chesnay, France; v Department of Intensive Care, Hospital Group of the Catholic Institute of Lille, FMMS-ETHICS EASaint Philibert Hospital, Catholic University of Lille, Lille, France; w Department of Medical Intensive Care, University Hospital of Ambroise Paré, Boulogne Billancourt, France; x Inserm U1018, Center for Research in Epidemiology and Population Health (CESP), Faculty of Paris Saclay, Villejuif, France; y Department of Intensive Care, Saint Louis Hospital, Paris, France; z Department of Intensive Care, Avignon Hospital, Avignon, France; A Department of Medical and Surgical Intensive Care, La Rochelle Saint Louis Hospital, La Rochelle, France; B University of Paris Cité, AP-HP, Hôpital Louis Mourier, DMU ESPRIT, Médecine Intensive Réanimation, Colombes, France and Université Paris Cité, INSERM, IAME, UMR1137, Paris, France; C Department of Medical and Surgical Intensive Care, Quimper Hospital, Quimper, France; D Department of Medical Intensive Care, Caen University Hospital, Caen, France; E Department of Medical Intensive Care, Edouard Herriot Hospital, Lyon, France; F Paris-Saclay Medical School, Paris-Saclay University, Le Kremlin-Bicêtre, France; G Institut für Anästhesiologische Pathophysiologie und Verfahrensentwicklung, Universitätsklinikum, Helmholtzstrasse 8-1, Ulm, Germany; H MITOVASC Laboratory UMR INSERM (French National Institute of Health and Medical Research), 1083 – CNRS 6015, University of Angers, Angers, France

**Keywords:** Evolution of core temperature, Hemodynamics, Hypothermia, Sepsis, Septic shock, Mortality

## Abstract

**Background:**

In patients with septic shock as well as in the critically ill, the impact of hypothermia and core temperature changes during the first 24 h on mortality remains uncertain. In this *post-hoc* analysis of the SEPSISPAM trial, we investigated the association between hypothermia at inclusion, hypothermia trajectories and 90-day mortality in patients with septic shock.

**Methods:**

This *post-hoc* analysis of the SEPSISPAM trial included patients with septic shock enrolled within 6 h of vasopressors initiation. Core temperature was assessed every 2 h during the first 24 h. Hypothermia was defined by a temperature <36 °C. Mortality was assessed at day 90.

**Results:**

We included 691 patients from the SEPSISPAM trial, of whom 103 (14.9%) presented with hypothermia at inclusion. After adjustment for confounding factors, as compared with patients without hypothermia at inclusion, patients with hypothermia at inclusion had a higher mortality (HR 1.92, 95% CI [1.38–2.67], p < 0.001). Three groups of patients were identified according to the evolution of their core temperature: “without hypothermia” (86.6%), i.e., patients without any hypothermia during the first 24 h; “transient hypothermia” (10%), i.e., patients with hypothermia at inclusion and becoming normothermic during the first 24 h, and “persistent hypothermia” (3.4%), i.e., patients with sustained hypothermia both at inclusion and during the first 24 h. Compared with patients without hypothermia, the “persistent hypothermia” group had the highest mortality rate at day 90 (78.3%, HR 2.83 [1.62−4.95], p < 0.0001). Mortality at day 90 increased according to temperature trajectories, being highest in patients with persistent hypothermia (78%), followed by those with transient hypothermia (49%), and lowest in patients without hypothermia (40%).

**Conclusion:**

In patients with septic shock, hypothermia at inclusion and persistence of hypothermia during the first 24 h were associated with higher mortality at day 90. Mortality increased according to the course of hypothermia during the first 24 h, being highest in patients with persistent hypothermia, followed by those with transient hypothermia, and lowest in patients who never developed hypothermia.

## Introduction

Bacterial infections and sepsis are usually associated with fever. However, recent data suggest that many critically ill patients with infection present with normal body temperature rather than fever [[1], [2], [3], [4]]. In addition, 9–21% of patients with sepsis present with hypothermia at admission to the intensive care unit (ICU) [[1], [2], [3], [4]], which, in turn, was associated with increased mortality [[5], [6], [7]]. Some data are available on the impact of hypothermia in patients with septic shock [8]. However, there is virtually no data on the relationship between the evolution of hypothermia during the first hours and mortality in this specific population. Septic shock is life-threatening with a high mortality rate [9]. Despite the frequency of temperature abnormalities, little research has been carried out on this subject, and a better knowledge may help to identify patients at higher risk of poor outcomes and support further research into temperature-related pathophysiological mechanisms and management strategies [3,4].

The multicenter randomized open-label trial SEPSISPAM recruited 776 patients at the early phase of the septic shock (i.e., within the 6 h following vasopressor introduction) [10]. Core temperature was recorded every two hours during the first 24 h of the trial period. Therefore, in this *post-hoc* analysis, we used data from the SEPSISPAM trial to investigate the impact of hypothermia at inclusion, as well as the evolution of the temperature during the first 24 h, respectively, on mortality in patients with septic shock.

## Materials and methods

### Ethical concerns

This *post-hoc* analysis concerns the SEPSISPAM trial (NCT01149278), which has obtained ethics approval from our University Hospital review board. Written informed consent was obtained from all patients, their next of kin or another surrogate decision-maker, as appropriate. In accordance with ethical policies, if patients were unable to provide informed consent and the next of kin or a designated person was not available, the emergency inclusion procedure was applied and *post-hoc* consent was obtained from patients who survived. In patients included under the emergency procedure who died before consent could be obtained and for whom no relative was available, data were retained for analysis in accordance with French legislation and ethic committee approval.

### Patient selection

In the randomized SEPSISPAM trial [10], adult patients (i.e., older than 18 years of age) were enrolled from 29 centers in France. Randomization was performed with the use of a computer-generated assignment sequence in a centralized, blinded fashion and was stratified according to whether patients had chronic arterial hypertension (i.e., had been receiving antihypertensive treatment or had a history of chronic arterial hypertension). Patients were enrolled if they had septic shock (according to the SEPSIS-2 definition [11]), if they required vasopressors (epinephrine or norepinephrine) at a minimum infusion rate of 0.1 μg per kilogram per minute, and if they had been evaluated within 6 h after the initiation of vasopressor. Exclusion criteria were legal protection (i.e, incompetence to provide consent and no guardianship or incarceration), no affiliation with the French health care system, pregnancy, recent participation in another biomedical study or another interventional study with mortality as the primary end point, or an investigator’s decision not to resuscitate. After enrollment, patients were assigned to vasopressor treatment adjusted to maintain a MAP of 80 to 85 mmHg (high-MAP target group) or 65 to 70 mmHg (low-MAP target group). The MAP target was maintained for a maximum of 5 days or until the patient was weaned from vasopressor support.

For the analysis about the relation between hypothermia at inclusion and mortality, we included patients with an available core temperature measurement at the time of inclusion. Then, for the analysis of temperature trajectories and time spent below the 36 °C threshold and mortality, we restricted the analysis to patients with at least six temperature measurements among the 13 scheduled time points (i.e., at inclusion and every two hours for 24 h). In other words, patients who died early during the first 24 h were excluded from the main analysis. A sensitivity analysis was subsequently performed including patients who died within the first 24 h despite having fewer than 6 temperature measurements. Missing data were not imputed. In the randomized SEPSISPAM trial, temperature management was left to the clinician's discretion, as no specific recommendations were available at that time [12].

### Data collection

In the SEPSISPAM trial, core temperature was measured according to protocol by nurses every 2 h from the inclusion to the first 24 h without specifying the method of measurement. Arterial lactate levels were measured at inclusion, 6 and 12 h after inclusion and once daily thereafter. The seasons were defined according to the calendar: Spring from March 21 to June 20, Summer from June 21 to September 20, Autumn from September 21 to December 20, Winter from December 21 to March 20.

### Outcomes

The primary outcome was to assess the relationship between hypothermia at inclusion and mortality up to 90 days. Hypothermia at the inclusion was defined as temperature below 36 °C at inclusion [11,13].

Secondary outcomes were to describe the evolution of temperature during the first 24 h and to assess the association between this temperature evolution and mortality up to day 90. Patient clusters were identified based on individual trajectory of temperature during the first 24 h of the septic shock.

### Temperatures

To describe the association between hypothermia and mortality, we successively considered the temperature at inclusion, longitudinal trajectories of temperature and the number of hours spent under 36 °C within the first 24 h. To study the trajectories of temperatures within the first 24 h, we identified clusters of patients with similar temperature trajectories using k-means group-based trajectory modeling [14]. This clustering analysis was restricted to patients who were hypothermic at inclusion (<36 °C) and the resulting clusters were compared with patients who were not hypothermic at inclusion. Model selection based on the Akaike Information Criterion (AIC) indicated that the 2-cluster solution provided the best fit for the longitudinal temperature trajectories within the first 24 h (lowest AIC value; Table S1). The 2-cluster solution, which had the lowest AIC value, also aligned well with clinical interpretation: one cluster corresponded to patients whose hypothermia resolved over time, whereas the other included patients with persistent hypothermia.

Three groups were isolated: (i) patients “without hypothermia” corresponding to patients without hypothermia during the first 24 h; (ii) patients with “transient hypothermia” corresponding to patients with hypothermia at inclusion, but in whom hypothermia had been corrected within the first 24 h; and iii) patients with “persistent hypothermia” corresponding to patients with hypothermia both at inclusion and persistent during the first 24 h.

### Statistical analysis

Quantitative variables, presented as median [interquartile range] were compared with Mann–Whitney test. Qualitative variables, presented as the absolute value [percentage] were compared with Chi 2 test. Censored variables were compared using log-rank test and presented using Kaplan Meier survival curves.

We used Cox regression models to explore the relation between hypothermia and mortality within 90 days. Results are presented as hazard ratio (HR) with 95% confidence intervals (95% CI) and p-value. In the first step, univariate analyses were conducted separately for each characteristic that differed between hypothermic and non-hypothermic patients at inclusion (which could represent potential confounding factors). To these criteria we added the presence of chronic corticosteroid treatment as a pre-existing condition, the use of corticosteroids at day 0, and the randomization group. In the second step, a multivariate Cox regression model was built, including all variables entered in the univariate analyses (full multivariate model).

Statistical analyses were performed using R (R Foundation for Statistical Computing, Vienna, Austria. URL https://www.R-project.org/) v4.4.3 and kml package [14]. No imputation of missing data was performed. All tests were two-sided, and p-values <0.05 were considered as statistically significant.

## Results

### Study population

**Fig. 1:**
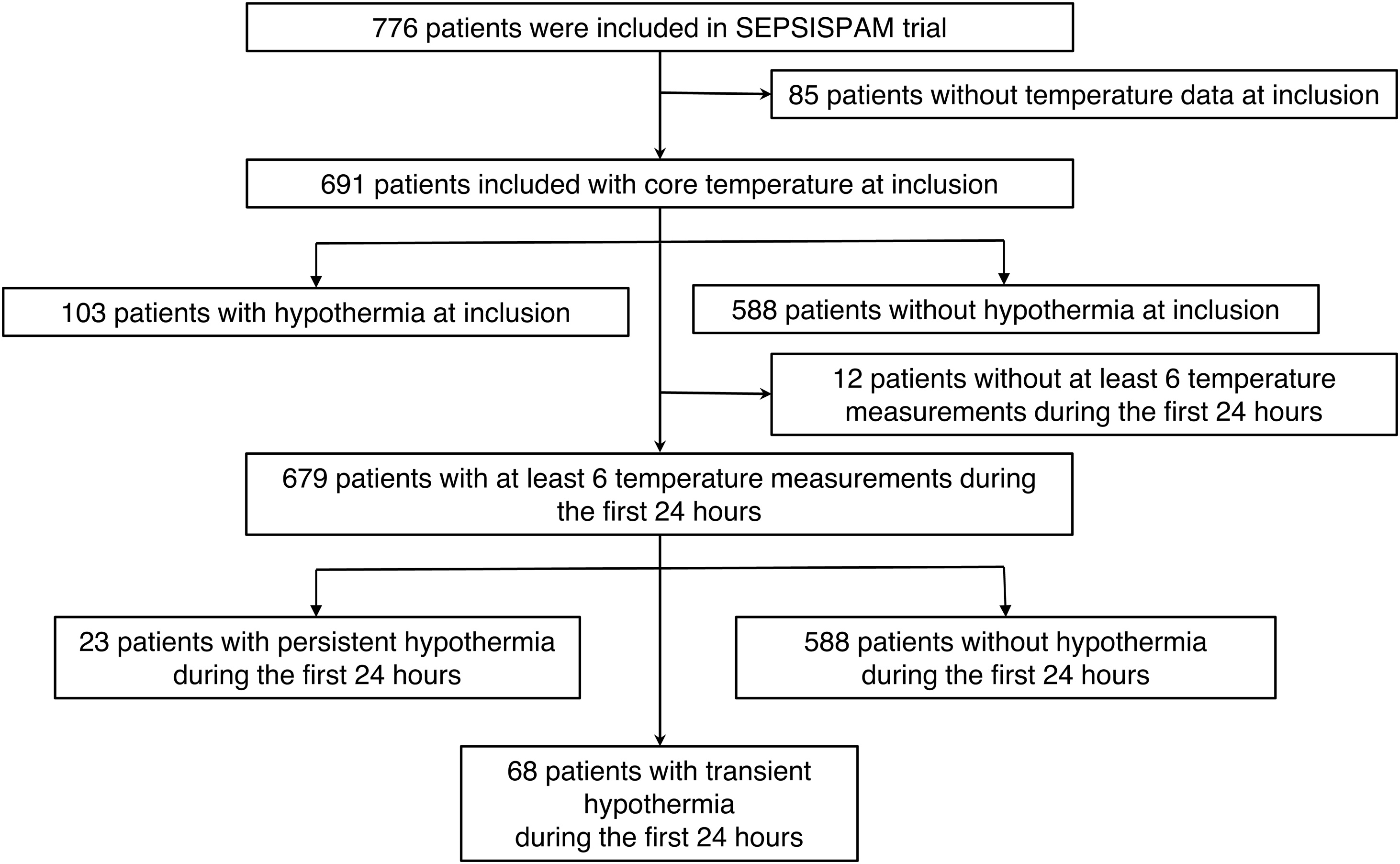
Flowchart of the study. SEPSISPAM trial refer to [10].

From 776 patients included in SEPSISPAM trial, 85 patients were excluded because of missing data regarding core temperature at inclusion, so that 691 patients were included in the primary outcome analysis (Fig. 1). Baseline characteristics of the 103 (14.9%) patients with hypothermia at inclusion and the 588 (85.1%) patients without hypothermia at inclusion are summarized in Table 1. Among patients with hypothermia at inclusion, 40 (39%) had a core temperature below 35 °C. Figure S1 shows the distribution of the temperature values at inclusion.

**Table 1:** Baseline characteristics of study patients according to the presence or absence of hypothermia at inclusion.

Characteristics	No hypothermia n = 588	Hypothermia n = 103	p-value
Age – years	65 [55–76]	69 [58–77]	0.087
Male sex - no (%)	394 [67]	67 [65]	0.7
Median temperature at inclusion - °C	37.40 [36.77–38.20]	35.20 [34.40–35.60]	**<0.001**
SAPS II	55 [44–65]	62 [53–77]	**<0.001**
SOFA	10.0 [8.0–12.0]	12.0 [10.0–14.0]	**<0.001**
Mechanical ventilation	439 [75]	90 [87]	**0.005**
PaO2/FiO_2_ ratio – mmHg	162 [109–250]	196 [121–272]	0.108
Acute kidney injury	255 [43]	67 [65]	**<0.001**

Pre-existing condition - no (%)			
Ischemic heart disease	60 [10]	15 [15]	0.2
Chronic heart failure	77 [13]	22 [21]	**0.027**
Chronic respiratory insufficiency	80 [14]	14 [14]	>0.9
Chronic kidney disease	37 [6.3]	8 [7.8]	0.6
Diabetes	123 [21]	25 [24]	0.4
Chronic arterial hypertension	286 [49]	57 [55]	0.2
Cirrhosis	37 [6.3]	11 [11]	0.11
Immunosuppression	174 [30]	15 [15]	**0.002**
Corticosteroids	83 [13]	6 [5.9]	**0.047**
Cancer or Autoimmune disease	218 [37]	30 [29]	0.129

Community-acquired infection	376 (64%)	79 (77%)	**0.012**
Source of infection - no (%)			
Lung	323 [55]	39 [38]	**0.002**
Abdomen	87 [15]	28 [27]	**0.002**
Urinary tract	65 [11]	12 [12]	0.9
Others	114 [19]	24 [23]	0.4

Pathogens - no (%)			
Gram+	180 [31]	33 [32]	0.8
Gram−	263 [45]	36 [35]	0.065
Yeasts	17 [2.9]	3 [2.9]	>0.9
Unidentified	177 [30]	42 [41]	**0.032**

Hemodynamic and biochemical variables			
Mean arterial pressure – mmHg	74 [65–83]	74 [64–82]	0.5
Heart rate - beats/min	103 [87–120]	90 [74–114]	**<0.001**
Mottling - no (%)	158 [27]	46 [46]	**<0.001**
Arterial lactate level - mmol/L	2.30 [1.40–3.60]	2.90 [1.63–7.18]	**<0.001**
Arterial pH	7.32 [7.23–7.39]	7.27 [7.14–7.37]	**<0.001**

Fluid therapy and vasoactive drug infusions			
Fluid therapy before inclusion – mL	2500 [2000–3500]	3000 [2500–3800]	**0.001**
Norepinephrine - no (%)	557 [95]	97 [94]	0.8
Dobutamine - no (%)	28 [4.8]	13 [13]	**0.002**
Epinephrine - no (%)	39 [6.6]	12 [12]	0.072
Median norepinephrine-equivalent vasopressor dose at inclusion - µg/kg/min *	0.34 [0.20−0.60]	0.40 [0.20−0.72]	0.3

Patients in the “No hypothermia” group had core temperature ≥36 °C at inclusion.

Patients in the “Hypothermia” group had core temperature <36 °C at inclusion.

Values are represented as median [interquartile range] or number (%) as appropriate.

The Simplified Acute Physiology Score (SAPS) II is based on 17 variables and scores range from 0 to 163, with a higher score indicating a more severe disease.

The score on the Sequential Organ Failure Assessment (SOFA) includes sub-scores ranging from 0 to 4 for each of the five components (circulation, lungs, liver, kidneys, neurological and coagulation). Aggregated scores range from 0 to 24, with higher scores indicating more severe organ failure.

Other sources of infection included blood, soft tissue, skin, central venous system, bones and joints, cardiac system, reproductive organs and unknown sources.

Acute kidney injury was defined as a renal SOFA score of 2 or more.

* Epinephrine doses were considered equivalent to norepinephrine doses on a dose-for-dose basis.

At inclusion, the median temperature of patients with hypothermia was 35.2 °C [34.4–35.6]. Compared with patients without hypothermia at inclusion, patients with hypothermia had higher disease severity, less frequent chronic corticosteroid treatment before inclusion and a higher incidence of chronic heart failure (Table 1). Patients with hypothermia at inclusion had higher mottling rates, lower arterial pH, higher arterial lactate levels, more frequent use of inotropic agents, and received larger amounts of resuscitation fluids prior to inclusion. As compared with patients without hypothermia at inclusion, the source of infection was more frequently abdominal and community-acquired in patients with hypothermia at inclusion. The proportion of gram-negative bacterial infections was similar in patients with and without hypothermia at inclusion. Compared with patients without hypothermia, the source of sepsis more often remained undetermined in patients with hypothermia at inclusion.

**Fig. 2:**
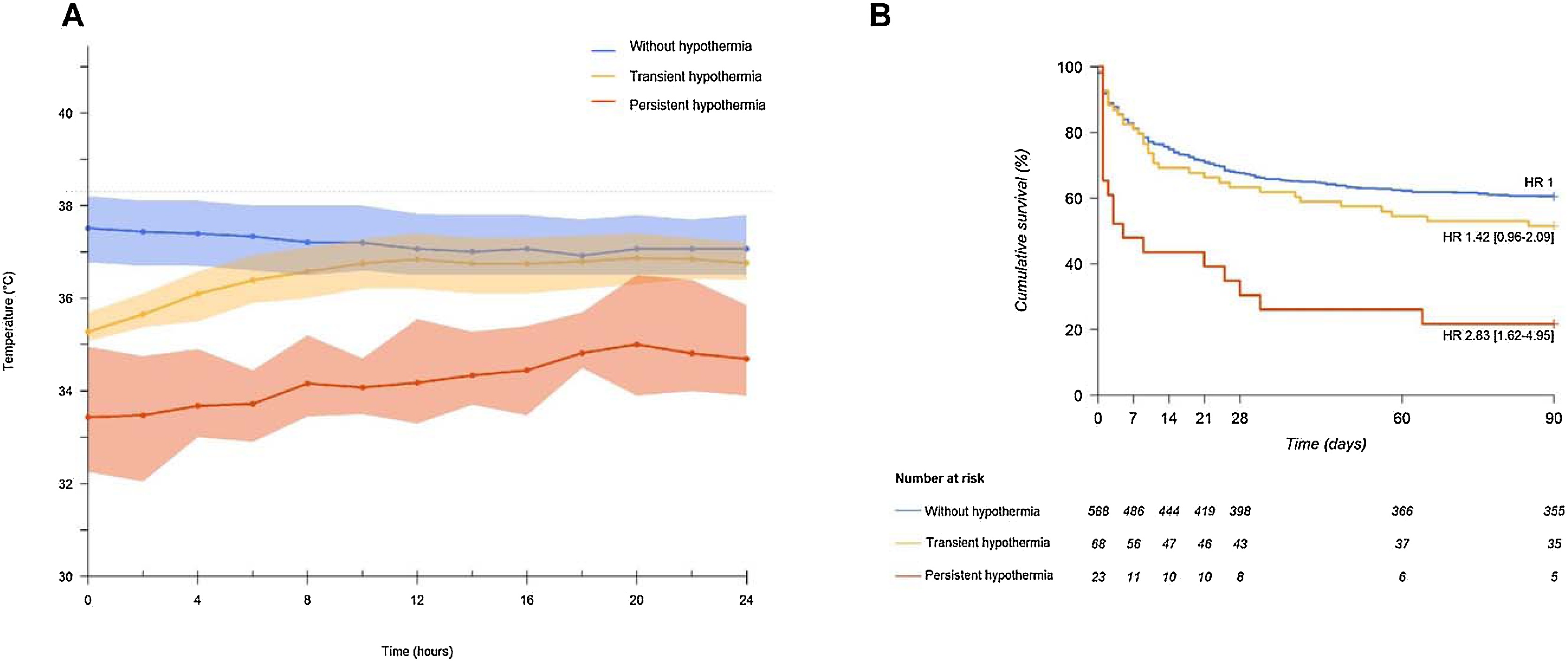
Clusters of patients according to the evolution of the core temperature during the first 24 h of the septic shock (A) and survival according to the course of the temperature (B). Patients “without hypothermia” corresponded to patients without hypothermia during the first 24 h; patients with “transient hypothermia” corresponded to patients with hypothermia at inclusion but whose hypothermia had corrected within the first 24 h. Patients with “persistent hypothermia” corresponded to patients with hypothermia at inclusion and whose hypothermia persisted during the first 24 h. Kaplan-Meier Curves represent the survival according to the course of temperature during the first 24 h of the septic shock. Hazard ratios (HR) were calculated using a Cox model. We defined the group of patients without hypothermia as the reference. HR were adjusted on SOFA, lactate, community-acquired infection, past medical history of cancer, and the presence of mottling at inclusion.

For the analysis of temperature trajectories over the first 24 h, only patients with at least 6 temperature measurements among the 13 scheduled time points were considered. A total of 679 patients were included among the 691 patients (Fig. 1). Among 12 patients with missing data during the first 24 h, all of them were hypothermic at inclusion and died during the first 24 h, 10 remained hypothermic until their death, 2 were considered with a transient hypothermia. 588 patients (86.6%) were in the group of patients “without hypothermia”, 68 patients (10%) in the group “transient hypothermia”, and 23 patients (3.4%) in the group “persistent hypothermia” (Fig. 2a).

Compared with patients “without hypothermia” or patients with “transient hypothermia”, patients with “persistent hypothermia” had higher severity scores and a higher proportion of mottling at inclusion (Table 2).

**Table 2:** Baseline characteristics of study patients in patients without hypothermia or with transient or persistent hypothermia during the first 24 h of the septic shock.

Characteristics	Without Hypothermia (n = 588)	Transient Hypothermia (n = 68)	Persistent hypothermia (n = 23)	p-value
Age – years	65 [55–76]	66 [57–76]	68 [60–77]	0.4
Male sex - no (%)	394 [67]	45 [66]	14 [61]	0.8
SAPS II	55 [44–65]	61 [50–72]	66 [54–78]	**<0.001**
SOFA	10.0 [8.0–12.0]	11.0 [9.0–13.0]	13.0 [11.0–14.0]	**0.003**
Mechanical ventilation - no (%)	439 [75]	59 [87]	20 [87]	**0.040**
PaO2/FiO_2_ ratio - mmHg	162 [109–250]	197 [130–271]	232 [141–352]	**0.042**
Acute kidney injury - no (%)	255 [43]	39 [57]	17 [74]	**0.002**

Pre-existing condition - no (%)				
Ischemic heart disease	60 [10]	6 [8.8]	3 [13]	0.8
Chronic heart failure	77 [13]	13 [19]	5 [22]	0.2
Chronic respiratory insufficiency	80 [14]	7 [10]	2 [8.7]	0.8
Chronic kidney disease	37 [6.3]	5 [7.4]	3 [13]	0.3
Diabetes	123 [21]	13 [19]	7 [30]	0.5
Chronic arterial hypertension	286 [49]	37 [54]	10 [43]	0.6
Cirrhosis	37 [6.3]	6 [8.8]	4 [17]	0.087
Immunosuppression	174 [30]	10 [15]	4 [17]	**0.018**
Corticosteroids during first 24 h	394 [68]	52 [79]	11 [48]	**0.020**
Cancer or Autoimmune disease	218 [37]	22 [32]	4 [17]	0.13

Community-acquired infection	376 [64]	50 [74]	18 [78]	0.12
Source of infection - no (%)				
Lung	322 [55]	26 [38]	9 [39]	**0.015**
Abdomen	87 [15]	17 [25]	7 [30]	**0.015**
Urinary tract	65 [11]	8 [12]	3 [13]	0.8
Others	114 [19]	17 [25]	4 [17]	0.5

Pathogens - no (%)				
Gram+	180 [31]	24 [35]	6 [26]	0.6
Gram−	263 [45]	25 [37]	8 [35]	0.3
Yeasts	17 [2.9]	3 [4.4]	0 [0]	0.6
Unidentified	177 [30]	24 [35]	11 [48]	0.15

Hemodynamic and biochemical variables				
Mean arterial pressure - mmHg	74 [65–83]	76 [64–82]	72 [66–82]	>0.9
Heart rate - beats/min	103 [87–120]	94 [77–115]	84 [73–102]	**0.001**
Mottling - no (%)	158 [27]	23 [35]	14 [61]	**0.001**
Serum lactate level - mmol/L	2.30 [1.40–3.60]	2.50 [1.60–5.10]	3.90 [1.50–12.80]	**0.041**
Arterial pH	7.32 [7.23–7.39]	7.28 [7.17–7.37]	7.26 [7.08–7.38]	**0.021**

Fluid therapy and vasoactive drug infusions				
Fluid therapy before inclusion - mL	2500 [2000–3500]	3000 [2500–4000]	3000 [2375–3625]	**0.013**
Norepinephrine - no (%)	557 [95]	66 [97]	21 [91]	0.4
Dobutamine - no (%)	28 [4.8]	7 [10]	3 [13]	**0.033**
Epinephrine - no (%)	39 [6.6]	4 [5.9]	4 [17]	0.14
Median norepinephrine-equivalent vasopressor dose at inclusion - µg/kg/min *	0.34 [0.20−0.60]	0.32 [0.20−0.71]	0.39 [0.22−0.65]	>0.9

Patients in the "without hypothermia" group corresponding to patients without hypothermia during the first 24 h.

Patients in the "transient hypothermia" corresponding to patients being normothermic during the first 24 h whereas they are hypothermic at inclusion.

Patients in the "persistent hypothermia" corresponding to hypothermic patients at inclusion and during the first 24 h.

Values are represented as median [interquartile range].

The target mean arterial pressure was 80–85 mmHg in the high-target group and 65–70 mmHg in the low-target group.

The Simplified Acute Physiology Score (SAPS) II is based on 17 variables and scores range from 0 to 163, with a higher score indicating a more severe disease.

The score on the Sequential Organ Failure Assessment (SOFA) includes sub-scores ranging from 0 to 4 for each of five components (circulation, lungs, liver, kidneys and coagulation). Aggregated scores range from 0 to 20, with higher scores indicating more severe organ failure.

Other sources of infection included blood, soft tissue, skin, central venous system, bones and joints, cardiac system, reproductive organs and unknown sources.

Acute kidney injury was defined as a renal SOFA score of 2 or more.

* Epinephrine doses were considered equivalent to norepinephrine doses on a dose-for-dose basis.

### Mortality at day 90

Mortality at day 90 was higher in patients with hypothermia at inclusion compared with those without hypothermia [63/103 (61%) vs. 232/588 (40%), p < 0.001]. Mortality at day 90 was higher in patients with persistent hypothermia compared with those with transient hypothermia or no hypothermia [18/23 (78%) vs 33/68 (49%) vs 232/588 (40%), respectively, p < 0.001] (Fig. 2b).

### Relation between hypothermia at inclusion and mortality

In univariate analysis (Table 3), the following factors were significantly associated with higher mortality within 90 days: hypothermia at inclusion, a higher SOFA score at inclusion, an arterial lactate level >2 mmol/L, tachycardia (i.e, heart rate >100 beats/min) at inclusion, nosocomial infection, history of chronic heart disease and the presence of mottling at inclusion. These analyses only included patients for whom complete data were available (n = 559). There were no differences between the patients included in this analysis and the overall study population (Table S2).

**Table 3:** Univariate and multivariate analysis for mortality at day 90 in patients with septic shock.

	Univariate Analysis†			Multivariate analysis†		
	n = 559			n = 559		
Variables	HR	95% CI	value	aHR	95% CI	value
Hypothermia (<36 °C vs 36−38.3 °C)	2.19	1.61–2.99	**<0.0001**	1.92	1.38–2.67	**0.0001**
Fever (>38.3 °C vs 36−38.3 °C)	1.23	0.85–1.77	0.27	1.08	0.74–1.59	0.6786
SOFA						
SOFA range 7 to 9	2.09	0.96–4.56	0.065	1.78	0.81–3.91	0.1459
SOFA range 10 to 12	2.62	1.21–5.67	**0.0158**	1.95	0.88–4.28	**0.0923**
SOFA range 13 to 14	3.60	1.62–8.01	**0.0017**	2.18	0.96–4.97	**0.058**
SOFA upper than 15	6.35	2.86–14.1	**<0.0001**	4.62	2.03–10.5	**0.0002**
Arterial lactate level						
No hyperlactatemia (<2 vs 2−4) mmol/L)	0.77	0.56–1.06	0.11	0.89	0.64–1.23	0.5392
Hyperlactatemia (>4 vs 2−4 mmol/L)	2.78	2.05–3.77	**<0.0001**	2.41	1.73–3.35	**<0.0001**
Without mottling at inclusion	0.43	0.33–0.55	**<0.0001**	0.54	0.41–0.71	**<0.0001**
Heart rate >100 bpm	1.01	1.00–1.01	**0.0363**	1.00	1.00–1.01	0.7679
Fluid intake upper than 1000 mL	1.03	0.94–1.13	0.5704	0.95	0.86–1.05	0.3112
Nosocomial infection	1.40	1.08–1.81	**0.0118**	1.70	1.27–2.26	**0.0003**
Gram negative	0.90	0.69–1.16	0.4086	0.97	0.74–1.26	0.7756
Cancer	1.18	0.91–1.54	0.2054	1.35	1.02–1.78	**0.0336**
Chronic heart disease	1.49	1.07–2.07	**0.0184**	1.28	0.90–1.82	0.1801
Corticotherapy before inclusion	1.18	0.82–1.70	0.3626	1.12	0.76–1.65	0.5675
Corticotherapy at day 0	1.13	0.85–1.49	0.3957	0.86	0.64–1.16	0.3176
Mean arterial pressure target (80−85 mmHg vs 65−70 mmHg)	0.90	0.70 – 1.16	0.4179	0.91	0.70–1.18	0.4846

Bpm: beats per minute. mL: millileters.

The univariate analysis included variables with p-value < 0.1 and clinical relevance. The multivariate model included all variable analyzed in the univariate analysis.

The score on the Sequential Organ Failure Assessment (SOFA) includes sub-scores ranging from 0 to 4 for each of five components (circulation, lungs, liver, kidneys, neurological and coagulation). Aggregated scores range from 0 to 24, with higher scores indicating more severe organ failure.

† Models presented are built using data from patients with no missing data for any of the variables studied.

Using multivariate analysis to adjust for confounding factors, patients with hypothermia at inclusion had a significant higher risk of death within 90 days than patients without hypothermia at inclusion (aHR 1.92, 95% CI [1,38–2,67], p < 0.001) (Table 3).

As an additional descriptive analysis, the severity of hypothermia was explored among patients presenting with a hypothermia at inclusion. Patients with hypothermia ≤35 °C at inclusion had a significantly higher mortality compared with those with mild hypothermia (35–36 °C) at inclusion (log-rank test, p = 0.03; Figure S2). Normothermic patients had a similar 90-day mortality compared with hyperthermic patients at inclusion (log-rank test, p = 0.59; Figure S3).

The seasons were not significantly associated with the presence of hypothermia at inclusion (Table S3).

In patients under 75 years of age, hypothermia at inclusion was associated with a significant increase in mortality (p < 0.001). Conversely, in older patients (i.e., >75 years), mortality rate was similar in patients with or without hypothermia at inclusion (p = 0.24) (Figure S4).

### Relation between the evolution of temperature during the first 24 h and outcomes

Compared with patients “without hypothermia” or with “transient hypothermia”, patients with “persistent hypothermia” had persistent hyperlactatemia (Figure S5) and, ultimately, higher mortality within the 90 days (Fig. 2b, log-rank p = 0.0001). Patients with “transient hypothermia” had lower mortality than those with persistent hypothermia, but higher mortality than patients without hypothermia (Fig. 2b). In a sensitivity analysis including the 12 patients who died during the first 24 h and therefore had incomplete temperature data, mortality remained highest in patients with persistent hypothermia (HR 4.04 [2.59–6.3], p < 0.0001 (Figure S6). In this analysis, patients with transient hypothermia also had a higher risk of death than patients without hypothermia (HR 1.6 [1.11–2.32], p = 0.0126) (Figure S6). The results are similar in patients with hyperlactatemia (>2 mmol/L) at inclusion (Figure S7a) and in those with lactate <2 mmol/L at inclusion (Figure S7b).

Among patients surviving 24 h after inclusion, any additional hour spent below the 36 °C threshold during the first 24 h was associated with an increased risk of 90-day mortality (HR 1.05 [1.03, 1.07], p = 0.0001). Hypothermia lasting for more than 14 h was associated with higher mortality at day 90 as compared to patients without hypothermia or with hypothermia lasting for up to 14 h (Figure S8).

## Discussion

This *post-hoc* analysis of the SEPSISPAM trial in patients with septic shock showed that hypothermia at inclusion and persistence of hypothermia during the first 24 h were associated with higher mortality at day 90 as compared with the absence of hypothermia. In addition, mortality differed according to temperature trajectories during the first 24 h: patients with persistent hypothermia had the highest mortality, whereas patients with transient hypothermia had lower mortality than those with persistent hypothermia, but higher mortality than patients without hypothermia.

Several authors have reported that hypothermia is associated with an increased risk of mortality in critically ill patients [15,16]. In patients with sepsis, hypothermia at admission was associated with higher mortality as compared with patients without hypothermia [6,7,17]. However, most of the studies were limited by the small numbers of patients included [18,19], and/or had been conducted in single or specialized ICUs [19,20]. In addition, a standard definition of “hypothermia” was lacking with variable thresholds ranging from 35 °C to 36.5 °C. Finally, very different timings of temperature recording, i.e., at admission or at any time within the first 24 h, were reported.

Several studies have reported an association between mortality and hypothermia in adult patients with sepsis. In patients with septic shock, two studies compared the mortality of patients with hypothermia at inclusion [21] (i.e., ≤35.6 °C) or at any time during their ICU stay [20] (i.e., ≤36 °C) with patients with hyperthermia (i.e., ≥38.3 °C). Both studies reported higher mortality in the presence of hypothermia. Furthermore, in a mixed population of patients with sepsis (n = 342) and septic shock (n = 282), occurrence of hypothermia (i.e., ≤36.5 °C) during the first 24 h was associated with higher mortality [2]. Similar results were found in a large population of patients with sepsis [22]. Finally, in a population of patients in an emergency department and requiring antibiotics for the management of infection, hypothermic patients (i.e., <36 °C) during the first days had a higher mortality than patients with normothermia or hyperthermia (i.e., >38 °C) [23].

So far, only one study [21] compared the impact of hypothermia at inclusion on the outcome of patients with septic shock. However, in this study, hypothermic patients (n = 195) were compared to hyperthermic patients (n = 735). Our study is the first to describe an association between mortality and the trajectory of hypothermia in patients with septic shock during the first 24 h of the ICU stay. Similar results were found in patients with sepsis and hypothermia (i.e., <36 °C) in emergency departments: an increase in core temperature of ≥1 °C during the initial 6 h was associated with a reduced 28-day mortality as compared to patients whose core temperature remained hypothermic [24]. Thus, in a well-defined population of patients with septic shock, our results show that mortality increases according to hypothermia trajectories during the first 24 h, being highest in patients with persistent hypothermia, lower in those with transient hypothermia, and lowest in patients without hypothermia.

Further studies are needed to determine whether hypothermia is a factor to be corrected during sepsis (rewarming) [3,4] or whether the spontaneous evolution of hypothermia is a factor associated with the correction of sepsis. Indeed, it is unclear whether hypothermia represents an abnormal response linked to the severity of the infection and a deregulation of the immune system [25] or whether it mirrors an adaptation to the severity of the septic shock to avoid the use of energy required to maintain normal core temperature [26]. The decrease in both pro-and anti-inflammatory responses in hypothermic patients with sepsis supports the theory of immune system deregulation in hypothermic patients with sepsis [22]. As for the alternative hypothesis of adaptation to the severity of septic shock, clinical studies have shown an increase in oxygen consumption during hyperthermia in patients with sepsis [27]. In contrast, hypothermia was associated with a decrease in oxygen consumption, potentially reducing metabolic demands [28]. Our *post-hoc* study does not allow us to answer this question.

In our study, arterial lactate levels, i.e., the biological hallmark of tissue hypoxia, were higher in patients with persistent hypothermia. The underlying mechanisms remain speculative; however, impaired cellular oxygen extraction due to altered mitochondrial respiration could lead to decreased oxygen consumption and reduced metabolic energy expenditure, ultimately resulting in hypothermia. Furthermore, an increase in pro-coagulation factors in patients with hypothermia during sepsis might exacerbate tissue hypoxia [22].

Our study has several limitations. First of all, the *post-hoc* design is well known for its biases. Second, we had no data on therapeutic temperature-control strategies, including the use of antipyretic drugs or active external warming devices, which may have influenced core temperature. In the SEPSISPAM study, no specific recommendations were provided regarding the management of hypothermia. Antipyretic agents, cooling maneuvers and warming blankets are routinely used in the ICU but were not recorded.

Moreover, in our trial, the method of measurement of temperature was not recorded (temperature measurement site, central or peripheral…), which so far, however, is a common observation in the other studies as well.

Third, the group of patients without hypothermia at inclusion was heterogeneous and included both normothermic and febrile patients. In our cohort, mortality did not significantly differ between normothermic and hyperthermic (≥38.2 °C) patients at inclusion. However, the present study was not designed to evaluate temperature trajectories specifically within febrile patients during the first 24 h. Future studies should explore temperature-based phenotypes more granularly, as different thermal profiles may reflect distinct biological and clinical septic phenotypes [29]. Recent interventional studies have suggested that both targeted cooling in hyperthermic patients [30] and therapeutic hyperthermia in afebrile septic patients may influence short-term outcomes [31], further supporting the need for a phenotype-driven approach to temperature management in sepsis.

Fourth, a standard definition of “hypothermia” is lacking. In this *post-hoc* analysis, only 40 patients had a core temperature at inclusion below 35 °C, which did not provide sufficient power to perform robust analyses using this more stringent threshold. However, our data suggest that hypothermia defined by a temperature < 35 °C at inclusion is also associated with increased mortality. The 36 °C threshold used in this study corresponds to the temperature criterion for the systemic inflammatory response syndrome definition [32]. Fifthalthough sensitivity analyses including patients who died early tended to accentuate the stepwise pattern of mortality across temperature trajectories, the relatively small sample sizes of the transient and persistent hypothermia groups limit the precision of effect estimates. Moreover, the relatively small size of the transient and persistent hypothermia subgroups precluded additional statistical approaches such as matching or propensity score analyses. As a result, residual confounding cannot be excluded, particularly given the greater severity observed in patients with persistent hypothermia. Thus, differences between adjacent groups should be interpreted with caution. Finally, the design of the SEPSISPAM trial and our *post-hoc* analysis did not allow to explain the pathophysiological relation between the evolution of body core temperature and mortality. Future experimental and clinical studies should further investigate biological parameters, including inflammatory biomarkers, cytokine profiles, heat and cold shock proteins, and metabolic markers, to better characterize the mechanisms linking hypothermia to mortality in sepsis and septic shock.

## Conclusion

In patients with septic shock, hypothermia at inclusion and persistence of hypothermia during the first 24 h were associated with higher mortality at day 90. The mortality increased stepwise according to the course of hypothermia during the first 24 h, being highest in patients with persistent hypothermia, followed by those with transient hypothermia, and lowest in patients who never developed hypothermia.

## Credit authorship contribution statement

FS, PA and NF designed the study. LB and NF collected data in hospitalization reports. LB and VS performed statistical analysis. LB, PA and NF drafted the article. FG, BM, NA, JPM, PFQ, SG, NW, FL, YLT, MC, RC, FG, CG, FT, JMT, JPB, TVDL, AVB, EM, GP, OL, JDR, FH, DDC, CG, AM, JLT and PA included patients in the SEPSISPAM trial and helped to grant access to hospitalization reports. All the authors have revised and approved the final version of the article.

## Consent for publication

Not applicable

## Ethics approval and consent to participate

The SEPSISPAM trial (NCT01149278) was approved for all participating centres by the ethic committee at the Angers University Hospital. Written inform consent was obtained from all patients, their next of kin or another surrogate decision maker, as appropriate. In accordance with ethics policy, if patients were unable to provide informed consent and the next of kin or a designated person was not available, the emergency inclusion procedure was applied and *post-hoc* consent was obtained it these latter patients.

## Funding

The SEPSISPAM trial was supported by the French Ministry of Health.

## Availability of data and materials

The datasets analyzed during the current study are available from the corresponding author on reasonable request.

## Declaration of competing interest

Dr Weiss received consultant fees from Med-Day Pharmaceuticals beyond the scope of this article.

Dr Dequin's institution received funding from Angers University Hospital, the French Ministry of Health, Abionic, Atox Bio, Sphingotec GMBH, Adrenomed, Medspace, Aridis, Merck, Combioxin, GSK, Med-Immune, Genentech INH, Rev-Immune, Faron, Kenta and Tigenix. He also received support for article research from the French Ministry of Health. Dr Gonzalez disclosed work for hire. Dr Teboul received funding from Getinge/Pulsion. Dr Radermacher's institution received funding from Deutsche Forschungsgemeinschaft and the German Ministry of Defence. The remaining authors have disclosed that they do not have any potential conflicts of interest.

## References

[1] Clemmer T.P., Fisher C.J., Bone R.C., Slotman G.J., Metz C.A., Thomas F.O. (1992). Hypothermia in the sepsis syndrome and clinical outcome. Crit Care Med..

[2] Kushimoto S., Gando S., Saitoh D., Mayumi T., Ogura H., Fujishima S. (2013). The impact of body temperature abnormalities on the disease severity and outcome in patients with severe sepsis: an analysis from a multicenter, prospective survey of severe sepsis. Crit Care..

[3] White K.C., Laupland K.B., Saxena M., Crichton B., McCullough J., Marella P. (2025). Sepsis in the absence of fever: determining the criteria for and feasibility of future therapeutic temperature management trials. Crit Care Resuscitation..

[4] Crichton B.B., Eathorne A., Coombes J., Edwards C., Falleni P.M., Laupland K.B. (2025). Temperature profiles in adult intensive care unit patients treated for infection in a tertiary intensive care unit: a single-centre prospective observational cohort study. Crit Care Resuscitation..

[5] Shimazui T., Nakada T., Walley K.R., Oshima T., Abe T., Ogura H. (2020). Significance of body temperature in elderly patients with sepsis. Crit Care..

[6] Thomas-Rüddel D.O., Hoffmann P., Schwarzkopf D., Scheer C., Bach F., Komann M. (2021). Fever and hypothermia represent two populations of sepsis patients and are associated with outside temperature. Crit Care..

[7] Kushimoto S., Abe T., Ogura H., Shiraishi A., Saitoh D., Fujishima S. (2019). Impact of body temperature abnormalities on the implementation of sepsis bundles and outcomes in patients with severe sepsis: a retrospective sub-analysis of the focused outcome research on emergency care for acute respiratory distress syndrome, sepsis and trauma study. Crit Care Med..

[8] Wu D., Lu S. (2020). The effects of abnormal body temperature on the prognosis of patients with septic shock. Ther Hypothermia Temp Manag..

[9] Annane D., Aegerter P., Jars-Guincestre M.C., Guidet B., CUB-Réa Network (2003). Current epidemiology of septic shock: the CUB-Réa Network. Am J Respir Crit Care Med..

[10] Asfar P., Meziani F., Hamel J.-F., Grelon F., Megarbane B., Anguel N. (2014). High versus low blood-pressure target in patients with septic shock. N Engl J Med..

[11] Levy M.M., Fink M.P., Marshall J.C., Abraham E., Angus D., Cook D. (2003). 2001 SCCM/ESICM/ACCP/ATS/SIS international sepsis definitions conference. Crit Care Med.

[12] Dellinger R.P., Carlet J.M., Masur H., Gerlach H., Calandra T., Cohen J. (2004). Surviving Sepsis Campaign guidelines for management of severe Sepsis and septic shock. Intensive Care Med..

[13] Singer M., Deutschman C.S., Seymour C.W., Shankar-Hari M., Annane D., Bauer M. (2016). The third international consensus definitions for sepsis and septic shock (Sepsis-3). JAMA..

[14] Genolini C., Alacoque X., Sentenac M., Arnaud C. (2015). **kml** and **kml3d**: *R* packages to cluster longitudinal data. J Stat Soft [Internet].

[15] Laupland K.B., Zahar J.-R., Adrie C., Schwebel C., Goldgran-Toledano D., Azoulay E. (2012). Determinants of temperature abnormalities and influence on outcome of critical illness. Crit Care Med..

[16] Young P.J., Saxena M., Beasley R., Bellomo R., Bailey M., Pilcher D. (2012). Early peak temperature and mortality in critically ill patients with or without infection. Intensive Care Med..

[17] Rumbus Z., Matics R., Hegyi P., Zsiboras C., Szabo I., Illes A. (2017). Fever is associated with reduced, hypothermia with increased mortality in septic patients: a meta-analysis of clinical trials. PLoS One.

[18] Brivet F., Carras P.M., Dormont J., Gidet B., Offenstadt G., Gachot (1994). Hypothermia, a pertinent clinical prognostic factor in severe systemic inflammatory response syndrome. Crit Care Med..

[19] Tiruvoipati R., Ong K., Gangopadhyay H., Arora S., Carney I., Botha J. (2010). Hypothermia predicts mortality in critically ill elderly patients with sepsis. BMC Geriatr..

[20] Peres Bota D., Lopes Ferreira F., Mélot C., Vincent J.L. (2004). Body temperature alterations in the critically ill. Intensive Care Med..

[21] Marik P.E., Zaloga G.P. (2000). Hypothermia and cytokines in septic shock. Intensive Care Med..

[22] Bhavani S.V., Spicer A., Sinha P., Malik A., Lopez-Espina C., Schmalz L. (2024). Distinct immune profiles and clinical outcomes in sepsis subphenotypes based on temperature trajectories. Intensive Care Med..

[23] Bhavani S.V., Carey K.A., Gilbert E.R., Afshar M., Verhoef P.A., Churpek M.M. (2019). Identifying novel sepsis subphenotypes using temperature trajectories. Am J Respir Crit Care Med..

[24] Han D., Kang S.H., Um Y.W., Kim H.E., Hwang J.E., Lee J.H. (2024). Temperature trajectories and mortality in hypothermic sepsis patients. Am J Emerg Med..

[25] Wiewel M.A., Harmon M.B., Van Vught L.A., Scicluna B.P., Hoogendijk A.J., Horn J. (2016). Risk factors, host response and outcome of hypothermic sepsis. Crit Care..

[26] Romanovsky A.A., Székely M. (1998). Fever and hypothermia: two adaptive thermoregulatory responses to systemic inflammation. Med Hypotheses..

[27] Manthous C.A., Hall J.B., Olson D., Singh M., Chatila W., Pohlman A. (1995). Effect of cooling on oxygen consumption in febrile critically ill patients. Am J Respir Crit Care Med..

[28] Goran M.I., Little R.A., Frayn K.N., Jones R., Fozzard G. (1988). Effects of chronic endotoxaemia on oxygen consumption at different ambient temperatures in the unanaesthetised rat. Circ Shock..

[29] Papathanakos G., Andrianopoulos I., Xenikakis M., Papathanasiou A., Koulenti D., Blot S. (2023). Clinical sepsis phenotypes in critically ill patients. Microorganisms..

[30] Schortgen F., Clabault K., Katsahian S., Devaquet J., Mercat A., Deye N. (2012). Fever control using external cooling in septic shock: a randomized controlled trial. Am J Respir Crit Care Med..

[31] Drewry A.M., Mohr N.M., Ablordeppey E.A., Dalton C.M., Doctor R.J., Fuller B.M. (2022). Therapeutic hyperthermia is associated with improved survival in afebrile critically ill patients with sepsis: a pilot randomized trial. Crit Care Med..

[32] Bone R.C., Balk R.A., Cerra F.B., Dellinger R.P., Fein A.M., Knaus W.A. (1992). Definitions for sepsis and organ failure and guidelines for the use of innovative therapies in sepsis. Chest..

