# Supplementary material for: Relation between initial hypothermia, course of the hypothermia and mortality in patients with septic shock: a post-hoc analysis of the SEPSISPAM randomized trial
Source: Ann Intensive Care. 2026 Mar 18;16:100051. doi: 10.1016/j.aicoj.2026.100051 (PMC13015728; doi:10.1016/j.aicoj.2026.100051)
Supplement: Supplementary file 1 [file mmc1.docx]

**SUPPLEMENTAL MATERIAL**

**Tables of contents**

[**SUPPLEMENTAL FIGURES** 2](#_Toc222585163)

[**Figure S1. Distribution of the core temperature at inclusion.** 2](#_Toc222585164)

[**Figure S2. Survival according to inclusion temperature categories (< 36°C, 36 – 38,1°C and ≥ 38,2°).** 2](#_Toc222585165)

[**Figure S3. Survival according to inclusion temperature categories (≥36°C, 35–36°C, and ≤35°C).** 2](#_Toc222585166)

[**Figure S4. Survival according to the presence or absence of hypothermia at inclusion in < 75 years old patients (A) and ≥ 75 years old patients (B).** 3](#_Toc222585167)

[**Figure S5. Evolution of the arterial lactate level in patients without hypothermia or with transient or persistent hypothermia during the first 24 hours of septic shock.** 3](#_Toc222585168)

[**Figure S6. Temperature trajectories during the first 24 hours of septic shock (A) and associated survival (B), including patients with early death (sensitivity analysis).** 3](#_Toc222585169)

[**Figure S7. Survival in patients with an arterial lactate level > 2 mmol/L at inclusion (A) and with an arterial lactate level < 2 mmol/L at inclusion (B) according to the course of the hypothermia during the first 24 hours of septic shock.** 4](#_Toc222585170)

[**Figure S8. Survival in patients who survived more than 24 hours according to the time spent below 36°C during the first 24 hours of septic shock.** 5](#_Toc222585171)

[**SUPPLEMENTAL TABLES** 6](#_Toc222585172)

[**Table S1. Number of clusters of longitudinal trajectories of temperature within the first 24 hours defined according to Akaike information criteria (AIC).** 6](#_Toc222585173)

[**Table S2. Characteristics used in the univariate and multivariate analysis, among patients included in the analysis and those excluded due to missing data.** 7](#_Toc222585174)

[**Table S3. Frequency of hypothermia at inclusion according to the seasons of the septic shock.** 8](#_Toc222585175)

# **SUPPLEMENTAL FIGURES**

## **Figure S1. Distribution of the core temperature at inclusion.**

*SEPSISPAM trial refers to [7].*

## **Figure S2. Survival according to inclusion temperature categories (< 36°C, 36 – 38,1°C and ≥ 38,2°).**

*Kaplan–Meier survival curves according to inclusion temperature categories: hypothermia (<36°C), normothermia (36–38.2°C), and hyperthermia (≥38.2°C). Overall survival differed significantly across the three groups (log-rank test p = 0.0001). In an additional descriptive comparison, patients with normothermia and hyperthermia at inclusion had similar 90-day mortality (log-rank test p = 0.59).*

## **Figure S3. Survival according to inclusion temperature categories (≥36°C, 35–36°C, and ≤35°C).**

*Kaplan–Meier survival curves according to inclusion temperature categories: no hypothermia (≥36°C), mild hypothermia (35–36°C), and moderate hypothermia (≤35°C). Overall survival differed significantly between the three groups (log-rank p < 0.001). In an additional descriptive comparison restricted to hypothermic patients, moderate hypothermia (≤35°C) was associated with higher mortality than mild hypothermia (35–36°C) (log-rank p = 0.03).*

## **Figure S4. Survival according to the presence or absence of hypothermia at inclusion in < 75 years old patients (A) and ≥ 75 years old patients (B).**

*The p-value refers to the log-rank test between patients without hypothermia at inclusion and with hypothermia at inclusion.*

## **Figure S5. Evolution of the arterial lactate level in patients without hypothermia or with transient or persistent hypothermia during the first 24 hours of septic shock.**

*The figure shows arterial lactate levels (in mmol/L) represented as median and interquartile range*

*Patients in the “without hypothermia” group corresponding to patients without hypothermia during the first 24 hours.*

*Patients in the “transient hypothermia” group corresponding to patients being normothermic during the first 24 hours whereas they are hypothermic (<36°C) at inclusion.*

*Patients in the "persistent hypothermia" corresponding to hypothermic patients at inclusion(<36°C) and during the first 24 hours.*

## **Figure S6. Temperature trajectories during the first 24 hours of septic shock (A) and associated survival (B), including patients with early death (sensitivity analysis).**

*Patients “without hypothermia” corresponded to patients without hypothermia during the first 24 hours; patients with “transient hypothermia” corresponded to patients with hypothermia at inclusion but whose hypothermia had corrected within the first 24 hours.*

*Patients with “persistent hypothermia” corresponded to patients with hypothermia at inclusion and whose hypothermia persisted during the first 24 hours.*

*Kaplan-Meier Curves represent the survival according to the course of temperature during the first 24 hours of the septic shock. Hazard ratios (HR) were calculated using a Cox model. We defined the group of patients without hypothermia as the reference. HR were adjusted on SOFA, lactate, community-acquired infection, past medical history of cancer, and the presence of mottling at inclusion.*

## **Figure S7. Survival in patients with an arterial lactate level > 2 mmol/L at inclusion (A) and with an arterial lactate level < 2 mmol/L at inclusion (B) according to the course of the hypothermia during the first 24 hours of septic shock.**

*The p-value refers to the log-rank test between patients “without hypothermia” corresponded to patients without hypothermia during the first 24 hours; patients with “transient hypothermia” corresponded to patients with hypothermia at inclusion but whose hypothermia had corrected within the first 24 hours; patients with “persistent hypothermia” corresponded to patients with hypothermia at inclusion and whose hypothermia persisted during the first 24 hours.*

## **Figure S8. Survival in patients who survived more than 24 hours according to the time spent below 36°C during the first 24 hours of septic shock.**

*The p-value refers to the log-rank test between patients without hypothermia during the first 24 hours, those with hypothermia for 2 to 12 hours, and those with hypothermia for more than 14 hours.*

# **SUPPLEMENTAL TABLES**

| **Number of clusters** | 2 | 3 | 4 | 5 | 6 |
| --- | --- | --- | --- | --- | --- |
| **AIC** | -3483.275 | -3197.739 | -3208.001 | -3225.816 | -3201.674 |

## **Table S1. Number of clusters of longitudinal trajectories of temperature within the first 24 hours defined according to Akaike information criteria (AIC).**

| **Variables** | | **With missing data**  **n = 197** | **Without missing data**  **n = 559** | **p-value** |
| --- | --- | --- | --- | --- |
| Température at inclusion - °C | | 37.3 (30 - 40.3) | 37.2 (30.3 - 40.4) | 0.179 |
| Randomisation group | MAP 80-85 mmHg  MAP 65-70 mmHg | 107/197 (54.3%)  90/197 (45.7%) | 269/559 (48.1%)  290/559 (51.9%) | 0.137 |
| Age – years | | 67 (20 - 92) | 66 (21 - 96) | 0.87 |
| SOFA range at inclusion | 0 to 6 | 6/136 (4.4%) | 38/559 (6.8%) | 0.652 |
|  | 7 to 9 | 41/136 (30.1%) | 178/559 (31.8%) | - |
|  | 10 to 12 | 52/136 (38.2%) | 192/559 (34.3%) | - |
|  | 13 to 14 | 24/136 (17.6%) | 84/559 (15%) | - |
|  | > 15 | 13/136 (9.6%) | 67/559 (12%) | - |
| Lactate range – mmol/L | 2 to 4 mmol/L | 37/124 (29.8%) | 210/559 (37.6%) | 0.203 |
|  | < 2 mmol/L | 53/124 (42.7%) | 226/559 (40.4%) | - |
|  | > 4 mmol/L | 34/124 (27.4%) | 123/559 (22%) | - |
| Heart rate – beats/min | | 99.5 (50 - 183) | 102 (52 - 204) | 0.108 |
| Fluid intake upper than 1000 mL – liter | | 2.5 (0 - 7.5) | 3 (0 - 10) | 0.068 |
| Nosocomiale infection | | 68/197 (34.5%) | 188/559 (33.6%) | 0.861 |
| Cancer | | 73/197 (37.1%) | 198/559 (35.4%) | 0.73 |
| Corticotherapy before inclusion | | 17/197 (8.6%) | 72/559 (12.9%) | 0.124 |
| Chronic heart disease | | 30/197 (15.2%) | 79/559 (14.1%) | 0.724 |
| Gram negative | | 87/197 (44.2%) | 245/559 (43.8%) | 0.934 |
| Mottling at inclusion | | 51/180 (28.3%) | 173/559 (30.9%) | 0.576 |
| Corticotherapy at day 0 | | 130/187 (69.5%) | 384/559 (68.7%) | 0.856 |

## **Table S2. Characteristics used in the univariate and multivariate analysis, among patients included in the analysis and those excluded due to missing data.**

*The score on the Sequential Organ Failure Assessment (SOFA) includes sub-scores ranging from 0 to 4 for each of five components (circulation, lungs, liver, kidneys and coagulation). Aggregated scores range from 0 to 20, with higher scores indicating more severe organ failure.*

|  | **No hypothermia**  **n = 588** | **Hypothermia**  **n = 103** | **p-value** |
| --- | --- | --- | --- |
| Winter | 106 (18%) | 17 (17%) | 0.7 |
| Spring | 155 (26%) | 29 (28%) | 0.7 |
| Summer | 173 (29%) | 33 (32%) | 0.6 |
| Autumn | 154 (26%) | 24 (23%) | 0.5 |
|  |  |  |  |

## **Table S3. Frequency of hypothermia at inclusion according to the seasons of the septic shock.**

*Spring was defined from March 20 to June 20, Summer from June 21 to September 22, Autumn from September 23 to December 21 and Winter from December 22 to March 19.*
